# Diagnostische Leistungsfähigkeit von zwei Antigentests im Vergleich zu einem PCR-Test zum Nachweis von SARS-CoV-2 in einer Notaufnahme und im Rettungsdienst

**DOI:** 10.1007/s00101-023-01343-8

**Published:** 2023-10-04

**Authors:** Veit Kinne, Sandra Ehrenberg, Michael Baier, Sebastian Lang, Jan-Christoph Lewejohann, Frank Kipp

**Affiliations:** 1 Institut für Infektionsmedizin und Krankenhaushygiene, Universitätsklinikum Jena, Jena, Deutschland; 2 Institut für Medizinische Mikrobiologie, Universitätsklinikum Jena, Jena, Deutschland; 3 Klinik für Anästhesiologie und Intensivmedizin, Universitätsklinikum Jena, Jena, Deutschland; 4 Klinik für Notfallmedizin, Universitätsklinikum Jena, Jena, Deutschland

**Keywords:** Krankenhaus, Pandemie, Prähospital, Point of Care Diagnostik, Klinik, COVID-19, Hospital, Pandemic, Prehospital, Point of Care Testing, Clinic, COVID-19

## Abstract

**Hintergrund:**

In der deutschen Krankenhauslandschaft sowie Notfallversorgung stellte die COVID-19-Pandemie eine Belastungsprobe dar. Die notfallmedizinische Gesundheitsversorgung wird in Deutschland durch die Versorgungskette zwischen prähospitaler Notfallrettung und klinischer Notfallversorgung in den Notaufnahmen sichergestellt. In Krankenhäusern und in der Notfallversorgung wird ein schneller, einfacher, genauer und kostengünstiger Test benötigt, um SARS-CoV‑2 zu identifizieren. In der zentralen Notaufnahme (ZNA) ist es wichtig, Patienten/Patientinnen mit COVID-19-Verdacht strikt von nichtinfizierten Notfallpersonen zu trennen.

**Methode:**

Es wurde die Leistungsfähigkeit von Antigentests in dem Rettungsdienst der Stadt Jena und in einer zentralen Notaufnahme des Universitätsklinikums Jena untersucht und mit dem RT-PCR-Goldstandard überprüft. Hierzu wurden multiple Testungen sowohl im Rettungswagen als auch in der Notaufnahme mittels Antigentests und RT-PCR-Tests durchgeführt. Insgesamt wurden 980 Patienten/Patientinnen in einem Zeitraum von 2 Monaten (Oktober und November 2022) in die Untersuchung eingeschlossen.

**Ergebnisse:**

Das Durchschnittsalter aller Patienten/Patientinnen lag bei 65 Jahren. Über die Hälfte der behandelten Patienten/Patientinnen kamen aus der Stadt Jena. Die Sensitivität und Spezifität der Antigentests lagen im klinischen Setting (in der Notaufnahme) bei 66,7 % resp. 99,2 % und im prähospitalen Setting (im Rettungsdienst) bei 68,8 % resp. 96,7 % im Vergleich zur RT-PCR. Im prähospitalen Setting lag die Sensitivität der Antigentests mit 2 % etwas höher als die klinische Antigentestung. Bei der Paralleltestung hatten 6 % der Antigentests ein falsch-negatives SARS-CoV-2-Antigentestergebnis im Rettungsdienst und 4,6 % der Antigentests ein falsch-negatives SARS-CoV-2-Antigentestergebnis in der ZNA. Die falsch-negativen Antigentests und somit die potenziell nicht erkannten Personen wurden durch die Betrachtung des Ct-Werts weiter abgeschätzt.

**Schlussfolgerung:**

Durch die Verwendung von Antigentests im Rettungsdienst und in der Notaufnahme kann eine zügigere Disposition in den COVID und Non-COVID-Bereich einer Notaufnahme erfolgen. Die Messgenauigkeit der Antigentests im Rettungsdienst und in der ZNA entspricht nicht der der RT-PCR. Dennoch ist der Antigentest ein nützliches Erstscreeninginstrument für die Früherkennung von SARS-CoV‑2 im prähospitalen und im klinischen Bereich. Eine zweifache Antigentestung kann für eine akkuratere Diagnostik des SARS-CoV-2-Erregers sinnvoll sein.

## Abstract (en)

**Background:**

In the German hospital landscape and emergency care the COVID-19 pandemic was a stress test. Emergency medical health care in Germany is ensured by the supply chain between prehospital emergency rescue and clinical emergency care in the emergency rooms. In hospitals and emergency care settings a rapid, simple, accurate, and cost-effective test is needed to identify SARS-CoV‑2. In the central emergency department it is important to strictly separate patients with suspected COVID-19 from non-infected emergency persons.

**Methods:**

Given the background mentioned above, the performance of antigen tests in the ambulance service of the city Jena and the central emergency department of the university hospital Jena was analysed and in addition verified by using the RT-PCR gold standard. Several multiple testing procedures were performed by using antigen tests in the ambulance service and the central emergency department, and by using one or both of these antigen tests followed by the RT-PCR test. A total of 980 patients were included in the study over a two-month period (October/November 2022).

**Results:**

The average age of all patients was 65 years. More than half of the actively treated patients came from the city of Jena. The sensitivity and specificity of the antigen tests were 66.7% and 99.2% in the clinical setting (the central emergency department) and 68.8% and 96.7% in the prehospital setting (in the ambulance service) compared to RT-PCR. In the prehospital setting the sensitivity of the antigen testing was slightly higher (2%) than the clinical antigen testing. Regarding the parallel testing, 6% of antigen tests had a false negative SARS-CoV‑2 antigen test result in the ambulance service and 4.6% of antigen tests had a false negative SARS-CoV‑2 antigen test result in the central emergency department. The false-negative antigen tests, and thus the potentially unrecognized individuals, were further reviewed by considering the Ct-value.

**Conclusion:**

The use of antigen testing in the ambulance service and the emergency department can lead to a quick classification of COVID and non-COVID areas of an emergency department. The measurement accuracy of antigen testing in the ambulance service and central emergency department is not equivalent to the RT-PCR. Nevertheless, antigen testing is a useful initial screening tool for early detection of SARS-CoV‑2 in prehospital and clinical settings. Dual antigen testing may be useful for more accurate diagnosis of the SARS-CoV‑2 pathogen.

## Einleitung

In der deutschen Krankenhauslandschaft sowie Notfallversorgung stellte die COVID-19-Pandemie eine Belastungsprobe dar. Das Coronavirus (SARS-CoV-2) breitete sich auch im Jahr 2022 weltweit aus und stellte die Gesundheitssysteme vor Herausforderungen. Die notfallmedizinische Gesundheitsversorgung wird in Deutschland durch die Versorgungskette zwischen prähospitaler Notfallrettung und klinischer Notfallversorgung in den Notaufnahmen sichergestellt. In der Notaufnahme kann es bei relativ kurzen Kontaktzeiten zu Transmissionen von Erregern (nicht nur SARS-CoV-2) und nosokomialen Infektionen kommen. Daher sollten Präventionsmaßnahmen, wie eine zeitnahe Identifikation und Isolation von infektiösen und nichtinfektiösen Personen, in Notaufnahmen zum Schutz der Patienten/Patientinnen etabliert sein [1–3]. Um dem gerecht zu werden, ist ein möglichst schneller, sensitiver und spezifischer Test essenziell.

Für die COVID-19-Diagnostik gilt als Goldstandard der molekularbiologische Nachweis in der Regel mittels Real-time Polymerase-chain-reaction (RT-PCR), bei der virale RNA nachgewiesen wird, unter Verwendung von Nasen-Rachen-Abstrich, Rachenabstrich oder Rachenspülwasser [2]. Die RT-PCR-Diagnostik ist jedoch material-, zeit- und kostenintensiv [3]. Dies bedingt, dass das Ergebnis eines regulären RT-PCR-Tests bei ambulant betreuten Patienten/Patientinnen häufig erst nach Beginn der Behandlung verfügbar ist und es oft zu Verzögerungen in Bezug auf die Verlegung in den Zielbereich kommt.

Eine wesentlich weniger material-, zeit- und kostenintensive Detektion kann über SARS-CoV-2-Antigen-Schnelltests (nachfolgend Antigentests) erfolgen, welche virale Proteine detektieren [3, 4]. Die Durchführung dieser Tests erfordert zudem kein Fachpersonal sowie keine spezielle Laborausstattung [5]. Professionelle Antigentests ermöglichen somit eine zeitnahe Identifizierung akut infizierter und potenziell infektiöser Personen und somit das Einleiten unmittelbarer Maßnahmen sowie die Optimierung interner klinischer Abläufe [6]. Antigentests werden häufig für ihre geringe Sensitivität und Spezifität beim Screening asymptomatischer Patienten kritisiert, dennoch besteht nach wie vor eine Wissenslücke hinsichtlich des Nutzens dieser Tests für das Screening [5]. Die in einem systematischen Review ermittelte Leistungsfähigkeit von Antigentests ergab eine nichtoptimale Sensitivität von durchschnittlich 56,2 %, konnte jedoch eine über 99 %ige durchschnittliche Spezifizität aufzeigen [7].

Im Herbst 2022 bestimmte die Coronavariante Omikron (BA.5) zu 93,9 % das Pandemiegeschehen in Deutschland [8]. Vor diesem Hintergrund und infolge begrenzter Erfahrungen mit Antigentests im prähospitalen und klinischen Setting wurden im Rahmen der vorliegenden Untersuchung Mehrfachtestungen mittels Antigen- und RT-PCR-Tests sowohl im Rettungstransportwagen (RTW) als auch in der zentralen Notaufnahme (ZNA) im Zeitraum von 2 Monaten durchgeführt und miteinander verglichen. In der Untersuchung sollten v. a. Erkenntnisse über die Sensitivität und Spezifität des Antigentests im Vergleich zur RT-PCR-Testung und die Möglichkeit einer seriellen Antigentestung zur Verbesserung der Detektion gewonnen werden. Darüber hinaus sollte geprüft werden, ob eine prähospitale Testung im Rettungsdienst den zweiten Antigentest in der ZNA ersetzen kann.

## Material und Methoden

**Figure 1:**
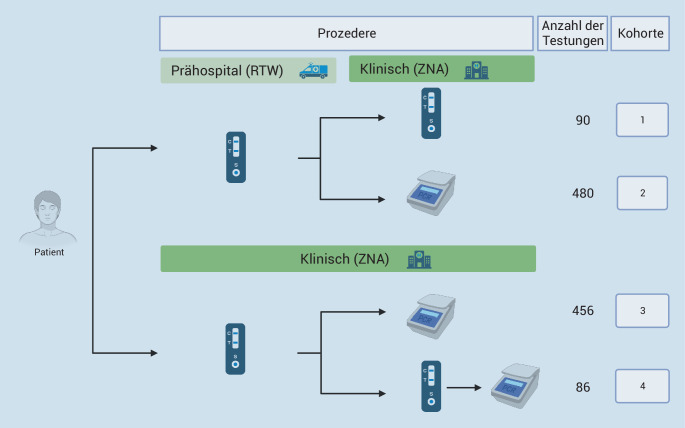

Im Zeitraum vom 01.10.2022 bis 30.11.2022 wurden insgesamt 980 Patienten/Patientinnen in die Untersuchung eingeschlossen. Innerhalb dieser Gruppe erfolgten 4 Testkohorten im prähospitalen (im RTW) und im klinischen (in der ZNA) Setting nach folgendem Muster (Abb. 1):

- 90 Testungen im prähospitalen und 90 Testungen im klinischen Setting mittels durchgeführten SARS-CoV-2-Antigentests (NanoRepro Corona Antigen Schnelltest, Fa. NanoRepro AG, Marburg, Deutschland), d. h. insgesamt 90 nacheinander durchführte Doppel-Antigentestungen im prähospitalen und klinischen Setting;
- 480 Antigentestungen im prähospitalen Setting und 480 RT-PCR Testungen im klinischen Setting, d. h. insgesamt 480 nacheinander durchgeführte Doppeltestungen mit jeweils Antigen- und RT-PCR Tests im prähospitalen und im klinischen Setting;
- 456 Antigentestungen und weitere 456 RT-PCR-Testungen im klinischen Setting, d. h. insgesamt 456 nacheinander durchgeführte Doppeltestungen mit jeweils Antigen- und RT-PCR-Tests im ausschließlich klinischen Setting;
- 86 Antigentestungen im prähospitalen, 86 Antigentestungen im klinischen und 86 RT-PCR-Testungen im klinischen Setting, d. h. insgesamt 86 nacheinander durchgeführte Dreifachtestungen mit 2‑mal Antigen- und einmal RT-PCR-Tests in prähospitalem und klinischem Setting (Abb. 1).

Die 4 Testkohorten wurden bei den in der Untersuchung eingeschlossenen 980 Patienten/Patientinnen durchgeführt. Eingeschlossen wurden dabei alle Patienten/Patientinnen, die im Untersuchungszeitraum durch den Rettungsdienst Jena und in der ZNA behandelt wurden und der Teilnahme an der Untersuchung zustimmten. Die Einteilung der Patienten/Patientinnen in die jeweiligen Testkohorten erfolgte randomisiert. Eine Aussage zu den COVID-19-Symptomen der Patienten/Patientinnen kann in der vorliegenden Untersuchung jedoch nicht getroffen werden.

Als Zuweiser beteiligte sich der Rettungsdienst in Jena an der vorgestellten Untersuchung. Bei Patienten/Patientinnen, die nicht über den Rettungsdienst in die ZNA eingeliefert wurden, wurde nur ein Antigentest in der ZNA durchgeführt.

Die Antigentestungen im RTW erfolgten durch das Rettungsdienstfachpersonal und in der ZNA durch das Pflegefachpersonal. Das Personal wurden im Vorfeld, basierend auf den Angaben des Testherstellers, über die Abnahmemodalitäten geschult. Die Auswertungen der prähospitalen und klinischen Antigentests erfolgten jeweils unmittelbar im RTW und in der ZNA. Die Testergebnisse des Antigentests lagen jeweils nach 15–20 min vor. Die Antigentests dienten ausschließlich zur Disposition der Patienten/Patientinnen in einen COVID- oder Non-COVID-Bereich der ZNA. Dazu wurde das prähospitale Testergebnis im Rettungsdiensteinsatzprotokoll dokumentiert. Für die Dokumentation in der ZNA wurde in der elektronischen Patientenakte eine COVID-19-Eingabemaske für prähospitale und klinische Antigentests eingerichtet.

In der ZNA wurde zeitgleich bei Abnahme des Antigenabstrichs auch ein RT-PCR-Abstrich vorgenommen. Im Rahmen des RT-PCR-Tests wurde im Labor des Universitätsklinikums Jena (UKJ) über eine zeitoptimierte molekularbiologische Methode (Cobas Liat, Fa. Roche Deutschland Holding GmbH, Freiburg, Deutschland) ein valides Ergebnis innerhalb von 20 min erzeugt. Jedes negative Testresultat wurde als Endergebnis unmittelbar freigegeben. Da der Liat-Test keinen Ct-Wert ausgibt, wurde jedes positive Resultat zur Bestimmung eines Ct-Wertes mit einem zweiten RT-PCR-System (NeuMoDx, Fa. Qiagen GmbH, Hilden, Deutschland) im Intervall bestätigt. Die positiven Ergebnisse des Cobas Liat (Fa. Roche) wurde dabei wie folgt in das NeuMoDx (Fa. Qiagen) überführt: Es wurden Aliquots des Originalmaterials aus dem eingesandten Originalröhrchen innerhalb von ca. 12 h im NeuMoDx nachgemessen. Vor der SARS-CoV-2-Pandemie stand bereits mindestens ein Liat-Gerät in der ZNA für die schnelle Influenzadiagnostik in der Saison zur Verfügung. Das Gerät wurde durch Mitarbeitende des Mikrobiologielabors betreut und vom Personal in der ZNA bedient.

Für die stationären Notfallpatienten wurden die Hauptdiagnosen ermittelt und aggregiert.

Für die deskriptive Statistik wurde eine Kreuztabelle verwendet. Für die Testkohorten wurde jeweils eine Detektionsrate (Anteil der positiven Antigentests im Rettungsdienst und der ZNA im Vergleich zum Anteil der positiven RT-PCR) berechnet. Die Detektionsrate kann auch als Positivrate verstanden werden. Sensitivität und Spezifität der verwendeten Methoden (Antigentests, RT-PCR) wurden berechnet. Für die Interpretation der Tests wurden der positive prädiktive Wert (PPW) und der negative prädiktive Wert (NPW) berechnet. Cohens κ wurde zur Berechnung der Interrater-Reliabilität verwendet (Testübereinstimmung). Die Auswertungen erfolgten mittels Microsoft Excel 2016 und SPSS Version 27 (IBM corp.). Die Fallnummern der Patienten/Patentinnen wurden im Vorfeld bereinigt und Duplikate entfernt.

## Ergebnisse

Das Durchschnittsalter der Patienten/Patientinnen lag bei 65 Jahren (Spannweite 18 bis 101 Jahre, *SD*  ± 21,28). 51,5 % waren männlich und 48,5 % weiblich.

Im Untersuchungszeitraum (Meldewochen 39 bis 48) wurde eine durchschnittliche Inzidenz von 340 auf 100.000 Einwohner für den Stadtkreis Jena ermittelt [9]. Es kamen 64,1 % (628) der behandelten Patienten/Patientinnen aus der Stadt Jena, gefolgt vom Saale-Holzland-Kreis mit 19,7 % (193) und Weimarer Land mit 2,0 % (20). Von den behandelten Personen wurden 53,1 % ambulant und 46,9 % stationär versorgt.

Von den 980 Patienten/Patientinnen waren insgesamt 166 Patienten/Patientinnen positiv auf SARS-CoV‑2 getestet. Im Hinblick auf die jeweils gebildeten Testkohorten lag die Detektionsrate bei 16,0 % (77/480) für die prähospitale Antigentestung und bei 19,3 % (93/480) für die korrespondierende RT-PCR-Testung. Die Sensitivität der Antigentestung im RTW im Vergleich zur RT-PCR-Testung in der ZNA betrug 68,8 %, die Spezifität 96,6 % (Tab. 1). Der PPW und der NPW der Antigentestung im RTW im Vergleich zur RT-PCR-Testung in der ZNA betrugen 83,1 % resp. 92,8 %. Cohens κ beträgt *κ* *=* 0,70; *p* *<* 0,001; 95 %-KI 0,62–0,78. Nach Landis und Koch (1977) repräsentiert das berechnete Cohens κ von 0,70 einen substanziellen Übereinstimmungswert für die beiden Tests im RTW und in der ZNA [10]. 42 Tests (8,75 %) wurden zwischen dem Antigentest im RTW und dem RT-PCR-Test in der ZNA falsch klassifiziert (Tab. 1). Hiervon waren 29 Antigentests falsch-negativ und 13 falsch-positiv.

**Table 1:** 

Testkohorte Antigentest im RTW im Vergleich zu RT-PCR (*n* = 480)		SARS-CoV-2-Antigentest im RTW			
		*Positiv*	*Negativ*	*Gesamt*	
	RT-PCR *positiv*	64	29	93	Sensitivität 68,8 %
	RT-PCR *negativ*	13	374	387	Spezifizität 96,6 %
Testkohorte Antigentest in ZNA im Vergleich zu RT-PCR (*n* = 456)		SARS-CoV-2-Antigentest in ZNA			
		*Positiv*	*Negativ*	*Gesamt*	
	RT-PCR *positiv*	42	21	63	Sensitivität 66,7 %
	RT-PCR *negativ*	3	390	393	Spezifizität 99,2 %

*RTW* Rettungstransportwagen, *ZNA* zentrale Notaufnahme, *RT-PCR* „real-time polymerase chain reaction“

Die Detektionsrate für die klinische Antigentestung lag bei 9,8 % (45/456) und 13,8 % (63/456) für die PCR-Testung. Die Sensitivität und Spezifität der Antigentestung im klinischen Bereich im Vergleich zur RT-PCR-Testung betrugen 66,7 % resp. 99,2 % (Tab. 1). Der PPW und der NPW der klinischen Antigentestung im Vergleich zur RT-PCR betrugen 93,3 % resp. 94,9 %. Cohens κ beträgt *κ* *=* 0,75; *p* *<* 0,001; 95 %-KI 0,66–0,85. Die Übereinstimmung mit Cohens κ ist somit statistisch signifikant. Das berechnete Cohens κ von 0,75 repräsentiert ebenfalls einen substanziellen Übereinstimmungswert für die beiden klinischen Tests [10]. Lediglich 24 Tests (5,2 %) wurden zwischen den Antigen- und RT-PCR-Test in der ZNA falsch klassifiziert. Hiervon waren 21 Antigentests falsch-negativ und 3 falsch-positiv (Tab. 1).

Bei der Antigentestung in der ZNA und im RTW lagen die Detektionsraten im direkten Vergleich bei 18,8 % (17/90) in der ZNA und bei 20 % (18/90) im RTW. Die Sensitivität und Spezifität des RTW-Tests gegenüber dem ZNA-Test (Standard) betrugen 99,4 % resp. 100 % (Tab. 2). Der PPW lag hier bei 100 % und der NPW bei 98,6 % (Tab. 2). Cohen’s κ beträgt *κ* *=* 0,96; *p* < 0,001; 95 %-KI 0,90–1,00. Das berechnete Cohens κ von 0,96 repräsentiert einen perfekten Übereinstimmungswert für die beiden Antigentests. Ein Antigentest im RTW wurde hier im Vergleich zur ZNA-Testung falsch klassifiziert (Tab. 2).

**Table 2:** 

Testkohorte Antigentest im RTW im Vergleich zu Antigentest in ZNA	SARS-CoV-2-Antigentest im RTW			
(*n* = 90)	Positiv	Negativ	Gesamt	
SARS-CoV-2-Antigentest in ZNA *positiv*	17	1	18	Sensitivität 94,4 %
SARS-CoV-2-Antigentest in ZNA *negativ*	0	72	72	Spezifizität 100 %

*RTW* Rettungstransportwagen, *ZNA* zentrale Notaufnahme, *RT-PCR* „real-time polymerase chain reaction“

Die Hauptdiagnosen der stationär behandelten Notfallpatienten/Notfallpatientinnen waren v. a. Krankheiten des Kreislaufsystems (24,4 %), Krankheiten des Atmungssystems (16,5 %), Verletzungen, Vergiftungen und bestimmte andere Folgen äußerer Ursachen (15,9 %) und Krankheiten des Verdauungssystems (9,7 %). Werden die Antigentests mit den aggregierten Hauptdiagnosen in Zusammenhang gebracht, so wurden bei der Diagnose Krankheiten des Atmungssystems 15 positive Tests bei der prähospitalen Antigentestung, 10 positive Antigentests in der ZNA und 26 positive RT-PCR-Tests festgestellt. Es wurde lediglich zwischen den positiven RT-PCR-Testungen und aggregierten Hauptdiagnosen ein schwacher statistischer Zusammenhang berechnet (χ^2^ = 0,363; *p* *=* 0,000; Cramers V = 0,225; *p* *=* 0,000). Die aggregierte Hauptdiagnose Krankheiten des Kreislaufsystems wurde an zweiter Stelle mit 8 positiven prähospitalen und 5 klinischen Antigentests sowie 11 positiven RT-PCR-Tests in Zusammenhang gebracht.

Bei Testungen, bei denen der prähospitale oder der klinische Antigentest negativ war und hingegen die RT-PCR positiv, wurde der Ct-Wert überprüft (falsch-negative SARS-CoV-2-Antigentestung), um einzuschätzen, in welchem semiquantitativen Mengenbereich die Antigentests eine bestehende Viruslast nicht erkannt haben. Es wurden insgesamt 50 Antigentests untersucht, die ein falsch-negatives SARS-CoV-2-Antigentestergebnis in der ZNA (*n* = 21) und im RTW (*n* = 29) im Vergleich zur RT-PCR-Testung aufwiesen (Tab. 3). Von den negativen SARS-CoV-2-Antigentests wurden in der RT-PCR-Laborbewertung insgesamt 33 RT-PCR-Tests als positiv und 17 Tests als schwach positiv eingestuft (Tab. 3).

**Table 3:** 

Prähospitale falsch-negative SARS-CoV-2-Antigentestung im Vergleich zur RT-PCR-Diagnostik		
*n* = 29	*Positiv*	*Schwach positiv*
Anzahl der RT-PCR	*19*	10
Ct-Wert	*M* *=* 25,62 (*SD* = 6,90)	*M* *=* 34,07 (*SD* = 6,79)
Klinische falsch-negative SARS-CoV-2-Antigentestung im Vergleich zur RT-PCR-Diagnostik		
*n* = 21	*Positiv*	*Schwach positiv*
Anzahl der RT-PCR	14	7
Ct-Wert	*M* = 24,66 (*SD* = 7,00)	*M* = 33,07 (*SD* = 6,47)

*Ct* „cycle threshold“, *ZNA* zentrale Notaufnahme, *RT-PCR* „real-time polymerase chain reaction“

Bei der Einzelbetrachtung der Antigentestergebnisse unabhängig von den Ergebnissen der Testkohorte aus Tab. 1 wurden 85 positive und 466 negative Antigentests im RTW (*n* = 551) und 54 positive und 462 negative in der ZNA (*n* = 516) festgestellt. Werden diese Einzelbetrachtungen den RT-PCR-Testungen gegenübergestellt, kann Folgendes konstatiert werden: Von den 85 positiven Antigentests im Rettungsdienst wies die RT-PCR im Nachgang insgesamt 64 Fälle (75 %) als SARS-CoV-2-Infektionen aus, und von den 54 positiven Antigentests in der ZNA wurden im Anschluss insgesamt 42 Fälle (77 %) durch die RT-PCR positiv getestet. Durch die Wiederholung des Antigentests in der ZNA hatte sich die Genauigkeit eines positiven Ergebnisses von 75 % auf 77 % leicht erhöht.

Eine Dreifachtestung (Antigentest RTW, Antigentest ZNA und RT-PCR) wurde bei 86 Patienten/Patientinnen durchgeführt. Hier wurden 17 positive und 69 negative Antigentests im RTW und 18 positive und 68 negative in der ZNA festgestellt. Bei der RT-PCR-Testung wurden 24 positive und 62 negative Testergebnisse festgestellt. Durch die Wiederholung des Antigentests in der ZNA wurde festgestellt, dass sich die Genauigkeit des positiven Ergebnisses von 19,8 % auf 20,9 % leicht verbesserte. Das positive Testergebnis des Antigentests in der ZNA im Vergleich zur RT-PCR-Testung stieg von 20,9 % auf 27,9 % (Tab. 4).

**Table 4:** 

SARS-CoV-2-Antigentest im RTW (*n* = 86)		SARS-CoV-2-Antigentest in der ZNA (*n* = 86)		RT-PCR-Test (*n* = 86)	
*Positiv*	*Negativ*	*Positiv*	*Negativ*	*Positiv*	*Negativ*
17 (19,8 %)	69 (80,2 %)	18 (20,9 %)	68 (79,1 %)	24 (27,9 %)	62 (72,1 %)

*RT-PCR* „real-time polymerase chain reaction“, *ZNA* zentrale Notaufnahme, *RTW* Rettungstransportwagen

## Diskussion

Durch eine schnelle, einfache und kostengünstige Anwendung sind Antigentests zur SARS-CoV-2-Detektion eine gute Ergänzung zur RT-PCR-Diagnostik im klinischen Setting [11]. Antigentests eignen sich als Erstscreeninginstrument im Rettungsdienst und in der Notaufnahme, um schnelle Interventionen einzuleiten und v. a. eine beschleunigte Disposition in den Zielbereichen zu erreichen. Dadurch kann eine weitere Transmission, ausgehend von infizierten Patienten/Patientinnen, im ambulanten und im stationären Bereich verhindert werden.

Zur Sensitivität und zur Spezifität des Antigentests der Fa. NanoRepro AG kann konstatiert werden, dass diese im klinischen Setting bei 66,7 % resp. 99,2 % und im prähospitalen Setting bei 68,8 % resp. 96,7 % im Vergleich zur RT-PCR lag. Ähnliche Ergebnisse wurden zu Sensitivität und Spezifität des Antigentests im Vergleich zum Goldstandard RT-PCR publiziert [3, 11–14]. Bezüglich der Leistung bzw. Performance der einzelnen Antigentests gibt es große Unterschiede [15–17]. Falsch-positive und falsch-negative Antigentestergebnisse können zu Kapazitätsengpässen führen oder das weitere Transmissionsgeschehen beeinflussen. Falsch-positive SARS-CoV-2-Ergebnisse treten ggf. bei fehlerhaften Tests und Kreuzreaktionen auf und gefährden Patienten/Patientinnen durch Kohortenbildung mit anderen COVID-19-Fällen. Häufige Ursachen für falsch-negative Antigentests sind: fehlerhafte Technik bei der Durchführung des Tests, unzureichende klinische Proben, Inhibitoren und Antigenabbau [18]. Gleichzeitig vorliegende andere Atemwegsinfektionen gefährden das Gesundheitspersonal und Patienten/Patientinnen bei nicht umfänglich diagnostizierten Erkrankungsfällen. Inwieweit die durch die falsch-negativen Antigentests potenziell nichterkannte Personen tatsächlich auch noch real infektiös sind, kann durch die Betrachtung des Ct-Werts abgeschätzt werden, wobei hier die Ansteckungsgefahr nur für die als „schwach-positiv“ deklarierten Fälle als gering eingestuft werden sollte (Tab. 3).

Im weiteren stationären Behandlungsverlauf sollte jedoch immer eine RT-PCR zur Bestätigung bzw. zum Ausschluss durchgeführt werden. Falls eine RT-PCR-Testung im stationären Setting nicht möglich ist, sollte auf eine repetitive Testung des Antigentests nach 2 bis 3 Tagen zurückgegriffen werden [19]. In Modellierungen konnte gezeigt werden, dass ein Screening alle 2 Tage mit einem schnellen, kostengünstigen und sogar wenig sensitiven (> 70 %) Point-of-care-Test (Antigentest) größere Ausbrüche in US-Hochschulen verhindern konnte [20]. Jedoch im klinischen Setting können die ermittelten Sensitivitäten < 70 % bei alleiniger Verwendung der Antigentestergebnisse eine Gefahr für das Patientenmanagement darstellen, da eine relevante Anzahl von infizierten Patienten/Patientinnen nicht erkannt wird. Vor allem in den vulnerablen Krankenhausbereichen, wie z. B. Onkologie, Hämatologie und Intensivmedizin, könnten dadurch vermehrte stationäre nosokomiale Ausbrüche entstehen. Daher sollte eine hochsensitive Methodik wie die RT-PCR an einer Schnittstelle wie der ZNA eine sinnvolle und verantwortliche Strategie sein und zum Einsatz kommen.

Eine Limitation der vorliegenden Untersuchung ist, dass die eingeschlossenen Patienten/Patientinnen nicht nach COVID-19-Symptomen differenziert wurden. Dadurch kann keine Aussage getroffen werden, inwiefern der verwendete Antigentest in der Notaufnahme und im Rettungsdienst spezifisch und sensitiv bei asymptomatischen sowie symptomatischen Personen ist. Eine weitere Limitation ist, dass keine detaillierte Aussage über die Viruslast getroffen werden kann.

Bislang gibt es nur wenige Untersuchungen darüber, welchen Nutzen ein prähospitaler Antigentest gegenüber dem klinischen Antigentest in der Notaufnahme bei der Aufnahme, Verteilung und Verlegung von Patienten/Patientinnen hat. Die vorliegenden Daten zeigen, dass im prähospitalen Setting die Sensitivität der Antigentests mit 2 % etwas höher lag als die klinische Antigentestung. Jedoch war die Spezifität im Gegensatz dazu ca. 2 % geringer. Eine prähospitale Antigentestung könnte v. a. bei anderen Erregern (z. B. Influenza) ein logistischer Vorteil für die Aufnahme und Behandlung von Patienten/Patientinnen in der Notaufnahme und im Krankenhaus bedeuten. Jedoch ist hier zu beachten, inwieweit eine prähospitale Antigentestung im Rettungsdienst überhaupt durchführbar ist, und welche logistischen Herausforderung diese mit sich bringt. Der Antigentest im Rettungsdienst wurde in der vorliegenden Untersuchung nur an einem Standort (Stadt Jena) durchgeführt. Im Untersuchungsort ist nur eine Klinik vorhanden, die von den Rettungsmitteln der Stadt Jena (5 RTW und 2 NEF/Notarzteinsatzfahrzeug) in der Regel angefahren wird. Die Logistik und Absprache waren entsprechend einfach im Vergleich zu Großstädten mit mehreren Kliniken und Rettungsdiensten aus mehreren Rettungsdienstbereichen. Für den Rettungsdienst hat die Antigentestung vor Ort auch hygienische und therapeutische Konsequenzen, da der Rettungsdienst seine Schutzausrüstung (Schutzkittel, Schutzoverall, Schutzbrille) erweitern muss, die Kontaktperson im Haushalt über die Isolationsmaßnahmen informiert wird und eine Reduzierung des Kontaktpersonals im RTW auf ein Minimum erfolgt. Darüber hinaus erfolgt bei Nachweis von SARS-CoV‑2 eine spezifische Fahrzeugdesinfektion. Bei einer erfolglosen Reanimation und positivem prähospitalen Antigentest muss der Notarzt/die Notärztin den Tod an das Gesundheitsamt melden. Daher sollte sich bei einer flächendeckenden Anwendung von prähospitalen und klinischen Antigentests im Rettungsdienst und in den Kliniken hinsichtlich Logistik, Finanzierung, Abnahmemodalität und Verwendung des gleichen Antigentests gut abstimmt werden. Des Weiteren sollte der Einsatz von Antigentests im Rettungsdienst nicht zu Verzögerungen in der prähospitalen Versorgung führen.

Neben den genannten logistischen Herausforderungen kann die Durchführung eines Antigentests bereits im Rettungswagen aber auch Vorteile haben. In einer retrospektiven Studie konnte mit einem Antigentest die Vorhersagegenauigkeit mit dem Goldstandard RT-PCR verglichen und von 38 % auf 92 % erhöht werden [5]. In der vorliegenden Untersuchung hatte sich durch die Wiederholung des Antigentests in der Notaufnahme die Genauigkeit gegenüber des positiven RT-PCR-Ergebnisses leicht verbessert. Weitere Studien zeigen, dass eine serielle Antigentestung selbst im nichtmedizinischen Setting eine genauere COVID-19-Detektion zur Folge hat [21, 22]. Durch diese Doppeltestung können die verminderte Sensitivität des Antigentests gegenüber der RT-PCR-Testung verbessert und falsch-negative Ergebnisse und deren Folgen reduziert werden [21].

Auch für künftige Erregerausbrüche oder Pandemien kann das Verfahren eines zweifachen Antigentests für eine akkuratere Diagnostik des Erregers sinnvoll sein, um Laborressourcen effektiver einzusetzen und Patienten schneller in entsprechende Zielbereiche zu verlagern.

## Fazit für die Praxis

- Die Verwendung von Antigentests im Rettungsdienst und in der Notaufnahme ermöglicht eine zügige Disposition in den COVID- und Non-COVID-Bereich einer Notaufnahme und reduziert das Transmissions- und Infektionsrisiko.
- Die Messgenauigkeit der Antigentests entspricht nicht der Messgenauigkeit der RT-PCR, jedoch kann die Antigentestung die Identifizierung von Patienten/Patientinnen im Rettungsdienst und in der Notaufnahme verbessern, v. a. wenn diese in Form einer Doppeltestung durchgeführt wird.
- Die Verlagerung des Testgeschehens erlaubt einen ressourceneffektiven Einsatz von Laborkapazitäten in Pandemiezeiten.
- Darüber hinaus wäre es vor dem Hintergrund aktuell abnehmender Inzidenzen von SARS-CoV‑2 künftig noch ressourceneffektiver, wenn Testungen ausschließlich bei symptomatischen Patienten/Patientinnen durchgeführt werden würden.
- Der Antigentest ist ein nützliches Erstscreeninginstrument für die Früherkennung von SARS-CoV‑2 im prähospitalen und klinischen Bereich, wobei die Durchführung im prähospitalen Bereich viele logistische Absprachen erfordert.

## Abkürzungen

NPW: Negativer prädiktiver Wert
PPW: Positiver prädiktiver Wert
RT-PCR: „Real-time polymerase chain reaction“
RTW: Rettungstransportwagen
UKJ: Universitätsklinikum Jena
ZNA: Zentrale Notaufnahme
